# Ready for Restitution? Meeting Challenges of Colonial Legacies in Africa's Collections

**DOI:** 10.1093/biosci/biaa167

**Published:** 2021-01-27

**Authors:** Solomon Sebuliba, Karsten Wesche, Willi E R Xylander

**Affiliations:** Senckenberg Museum, Natural History Goerlitz, Goerlitz, Germany, and with the International Institute Zittau, Technische Universität Dresden, Zittau, Germany; Senckenberg Museum, Natural History Goerlitz, Goerlitz, Germany, and with the International Institute Zittau, Technische Universität Dresden, Zittau, Germany; Senckenberg Museum, Natural History Goerlitz, Goerlitz, Germany, and with the International Institute Zittau, Technische Universität Dresden, Zittau, Germany

Movements have gained momentum within the restitution debate with the aim of taking cultural objects back to Africa. The discussions are vivid. For instance, the Savoy Report stated that Europe's museums keep an overwhelmingly large share of Africa's collections, leaving the countries of origin with almost nothing (Sarr and Savoy 2018). Indeed, most—if not all—large museums in Europe and North America bear African collections with colonial legacies. Either their specimens originated directly from former colonies or they were obtained in the context of questionable international procedures put in place at the time. As a consequence, the content, interpretation, and representation of indigenous cultural entities in museums are contentious issues of discussion (Ashley 2009). Undoubtedly, Africa has become a prime region for discussing colonial legacies. It is a debate about moral and power pressure (Shyllon 2017).

Natural history collections had not previously been the focus of respective discussions, but this is now changing. Debates about the repatriation of human remains (e.g., Smith 2004) and restitution of biological, palaeontological, and geological objects to countries of origin are increasing (e.g., Curtis 2006). We have the utmost respect for such opinions. No museum or collection-holding institution should ignore these claims. However, we believe that there are more facets than are presently being discussed. In the present viewpoint, we briefly list some of these and add new empirical data for one specific aspect.

Human remains and cultural artifacts made by local people should be subjected to specific high ethical standards, because they constitute national and regional cultural heritage; they may therefore create an identity for the people, *sensu stricto* (Camara 2014). Repatriation on demand—for example, to bury human remains or for restitution of outstanding peoples’ cultural objects—should be considered a high priority. However, elements of recent or extinct flora (in the broader sense) and fauna or geology have a different ontological status, given that we can also view nature as humanity's shared heritage.

## The state of natural history collections: Empirical data from a survey in East Africa

There are around 1.5 billion specimens in Europe's natural history collections alone (for an overview, see https://dissco.eu), and a small share of these can be considered culturally sensitive in a stricter sense. The remainder also bears legacies of colonial times, but general claims for restitution convey not only theoretical but practical problems. The key questions include what may happen after restitution. Who will be responsible, for instance, for the accessibility of research, taxonomic reference, maintenance, and curation and for the cost? And is this the most urgent need for Africa's museums? Many of the answers depend on the state of the potential receiver institutions, but, unfortunately, the documentation of Africa's museums is principally deficient or even totally lacking; we know little about these institutions (Sebuliba 2020).

Therefore, we conducted a survey of natural history museums and ethnographic collections in East Africa (Uganda, Rwanda, and the National Museum of Kenya (Sebuliba 2020). We made structured face-to-face interviews with, for example, museum staff and visitors and made our assessments, discovering several opportunities but also critical challenges these institutions are facing. On the basis of these interviews, we scored the museums against a set of key performance indicators. We also asked the museum leads to provide a self-assessment of those scores. They were, in most cases, less critical (figure 5a of Sebuliba 2020)—but only slightly. Therefore, we report our assessments in the present article.

**Figure 1.**
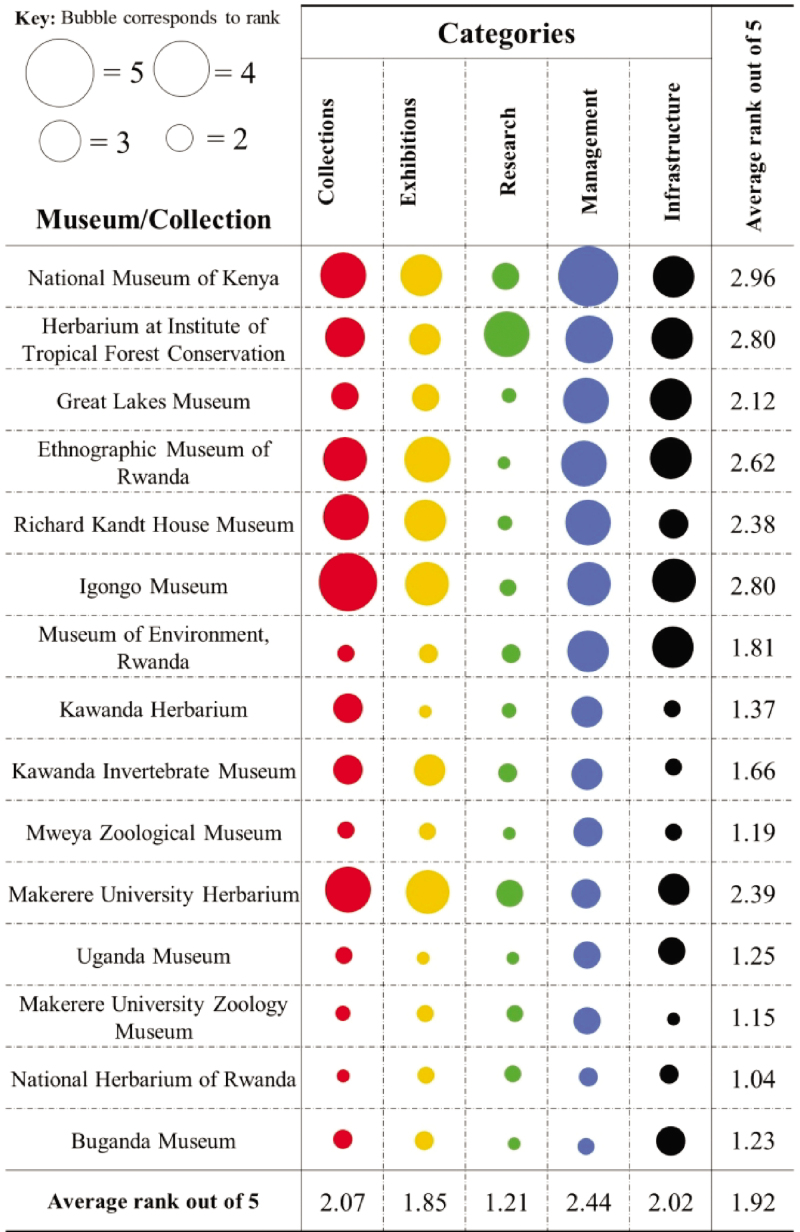
Assessment of categories using Smithsonian quality ratings for all museums or collections assessed. Categories were divided into different indicators defining standards, on which basis they were rated. Quality criteria: 5, very good (all indicators present and in a satisfactory state); 4, good (many indicators satisfactory; improvements are needed but may not be urgent); 3, just good (several indicators are satisfactory, but many need urgent improvements); 2, low quality (most indicators are in a poor state with urgent improvements needed); 1, very low quality (all most all indicators are in a poor state with urgent improvements needed; many indicators may also be absent). The data are from Sebuliba (2020).

Apart from a few large museums that are relatively well managed, such as the National Museum of Kenya, most natural history collections are in a poor state, with financial support essentially lacking. Among other challenges, curation is lacking; there are no proper infrastructures (buildings, labs); and many lack expertise, including scientific and technical staff (e.g., trained curators; figure 1). Most institutions scored as low or very low quality regarding all of the aspects we assessed. Proper facilities, including maintenance, prevention, and taxonomic experts and their active research, are, however, key for maintaining the scientific value of collections. Recent tragic losses, such as the case of the National Museum in Brazil (Kury et al. 2018), highlight that even well maintained institutions are at risk. Those under poor management are even more so (Sebuliba 2020).

Many African countries, currently stand at risk of failure to maintain their collections and of not using them for their intended scientific and educational purposes. The internal views of the museums assessed in the survey reflect these finding. They generally do not regard restitution as an urgent need but, rather, advocate a significant improvement of infrastructure and expertise (more recommendations; Sebuliba 2020).

## A way out? Reflections on the restitution debate

Museums in Africa have huge collections, including many unique objects, and, still, much of these collections has not yet been sampled, documented, researched, or investigated on the continent. The notion that most of Africa's heritage is in Europe seems an exaggeration concerning natural history collections. Rebalancing the geography of African cultural collection disposition across the world, as was suggested in the Savoy Report (Sarr and Savoy 2018), must be considered but needs a much broader scope.

Is restitution intended to restore the “lost” African heritage and to correct earlier mistakes? If so, the efforts should be primarily focused on reinstating and, in many cases, boosting the integrity of the systems and institutions that are now responsible for maintaining heritage in Africa. Many institutions got dismantled during and after the colonial period, and those presently responsible are struggling to sustain even those objects they hold (Sebuliba 2020). The respective measures can range from infrastructure improvement in terms of buildings, building up lab facilities, or installing state-of-the-art storage systems. Building research capacity is an even more pressing need for the continent's museums (figure 1). First and foremost, there is a need to develop human resources; therefore, courses on the taxonomy and management of natural history collections are a fundamental element, but only a few of these are available, such as specialized MS programs (e.g., BCM 2020). Organizing the respective scholarships is another essential element, and involving African scholars in the international scientific exchange is another. Digital methods offer unprecedented opportunities here (Arts et al. 2015).

## Conclusions and a plea for a differentiated debate

Human remains and specific outstanding cultural artifacts are a priority for restitutions on demand, and museums do have a high responsibility here. Even so, taking back objects should always be accomplished by ensuring properly curated facilities that guarantee state-of-the-art maintenance, access for the world's scientific community (in the museum, as well via freely available databases), and high-quality research facilities. Consequently, all of these decisions become far more important when it comes to the vast numbers of other biological, paleontological, and geological specimens gathered in the context of colonialism. The governments and the collections institutions of former colonial powers should work together with partners in Africa to assess the current needs and to jointly develop targeted solutions. They can build on several strategies, such as a physical exchange of representative material, such as paratypes. Other approaches may include object digitization and imaging or improved working conditions through infrastructure investments.

More importantly, science cooperation in the broadest sense is crucial. Restitution should therefore be seen as one element among others, and we fully respect countries that have requested to return specific objects. We believe they understand their responsibility, their commitment, and the maintenance costs that come with collections. However, the entire debate is rooted in a remarkably complex colonial history, and the solutions to overcome that legacy need to be elaborate too. Improving the quality and relevance of Africa's museum collections to society, as well as ensuring that worldwide museums and collections stay in contact with easy access to data and material, is a much more comprehensive aim and is in the best interest of all those involved (Sebuliba 2020).

## Author Biographical

Solomon Sebuliba (nsaxon99@gmail.com), Karsten Wesche, and Willi E. R. Xylander are affiliated with the Senckenberg Museum of Natural History Goerlitz, in Goerlitz, Germany, and with the International Institute Zittau, at Technische Universität Dresden, in Zittau, Germany.
